# Physical activity, health-related quality of life, and perceived barriers among the Alfaisal University Community in Riyadh, Saudi Arabia: a cross-sectional study

**DOI:** 10.3389/fpubh.2026.1999364

**Published:** 2026-10-23

**Authors:** Yara O. Alsouss, Omar Z. Ameer, Ghaidaa A. Alfaraj, Peter M. B. Cahusac, Abdullah Ibrahim Al Sharif, Noara Alhusseini

**Affiliations:** 1 Department of Biostatistics, Epidemiology and Public Health, College of Medicine, Alfaisal University, Riyadh, Saudi Arabia; 2 Department of Pharmaceutical Sciences, College of Pharmacy, Alfaisal University, Riyadh, Saudi Arabia; 3 Jazan Health Cluster, Jazan, Saudi Arabia; 4 Department of Pharmacology & Biostatistics, College of Medicine, Alfaisal University, Riyadh, Saudi Arabia

**Keywords:** EQ-5D-5L, health-related quality of life, IPAQ-SF, perceived barriers, physical activity, university population

## Abstract

Physical activity is an important component of health promotion, yet its relationship with health-related quality of life (HRQoL) within university populations in Saudi Arabia remains insufficiently understood. This cross-sectional study examined physical activity, HRQoL, and perceived barriers to physical activity among 353 students, faculty, and staff at Alfaisal University in Riyadh, Saudi Arabia. Physical activity was assessed using the International Physical Activity Questionnaire–Short Form, HRQoL using the EQ-5D-5L and EQ-VAS, and perceived barriers using the Barriers to Being Active Quiz. Multivariable regression models were used to examine associations after adjustment for sociodemographic and health-related factors. Moderate physical activity was the most frequently reported activity category. Physical activity level was not significantly associated with either HRQoL outcome after adjustment. Compared with low physical activity, high physical activity was associated with neither EQ-VAS (B = 4.055, BCa 95% CI: −2.082 to 10.426, *p* = 0.197) nor EQ-5D-5L index scores (B = −0.005, BCa 95% CI: −0.035 to 0.028, *p* = 0.824). Pre-existing mental health conditions were associated with lower EQ-VAS (B = −11.891, *p* = 0.010) and index scores (B = −0.088, *p* = 0.001), while pre-existing physical conditions were associated with lower index scores (B = −0.042, *p* = 0.013). Female participants had higher odds of being in a lower physical activity category than males (adjusted OR = 1.897, 95% CI: 1.267–2.840, *p* = 0.002), whereas Saudi participants had lower odds of being in a lower physical activity category than non-Saudi participants (adjusted OR = 0.589, 95% CI: 0.396–0.875, *p* = 0.009). Lack of time, energy, and willpower were the most frequently reported barriers to physical activity. These findings suggest that, within this predominantly young academic population, underlying health status may be an important correlate of HRQoL, whereas physical activity level was not independently associated with HRQoL. University health promotion strategies may therefore benefit from integrating physical activity initiatives with mental health support and interventions addressing common behavioral barriers.

## 1 Introduction

Health-related quality of life (HRQoL) is a multidimensional measure of perceived physical and mental health that captures how health influences daily functioning and well-being (1, 2). Beyond conventional clinical outcomes, HRQoL provides a patient-reported perspective that can inform population health assessment, evaluation of health interventions, and healthcare decision-making (2). Physical activity is similarly an important component of population health. Regular physical activity contributes to the prevention and management of noncommunicable diseases and is associated with better physical and psychological well-being, whereas insufficient physical activity remains a substantial contributor to morbidity and mortality worldwide (3, 4). In Saudi Arabia, promoting active lifestyles and improving population well-being have become increasingly relevant within the broader health transformation agenda of Saudi Vision 2030 (5).

University communities represent an important setting in which to examine these outcomes. Academic environments can involve prolonged sitting, demanding study or work schedules, and competing responsibilities that may influence opportunities for physical activity. A meta-analysis of university students reported substantial sedentary time, averaging 7.29 h per day based on self-reported measures and 9.82 h per day when objectively measured (6). Similar concerns have been reported in Saudi universities, where high levels of sedentary behavior coexist with considerable variation in physical activity participation (7–9). These patterns are particularly relevant because university environments include not only students but also faculty and staff, whose academic and occupational demands may differ while sharing the same institutional environment.

International evidence generally suggests a positive relationship between physical activity and HRQoL, although the magnitude and consistency of this association vary across populations and measurement approaches. A systematic review and meta-analysis found that physical exercise was associated with improvements in general and mental HRQoL among office workers, with particularly notable benefits among individuals with pre-existing health conditions (10). Positive associations between physical activity and quality-of-life outcomes have also been reported among university students (11). However, HRQoL in academic populations is influenced by multiple factors beyond physical activity. Mental health, chronic health conditions, sedentary behavior, and academic or occupational pressures may independently influence perceived health and well-being (12–14).

Evidence from Saudi university populations has primarily focused on physical activity prevalence, sedentary behavior, or barriers to participation, with limited evidence examining physical activity and HRQoL together within a broader university community. Studies have identified substantial sedentary time and variable physical activity levels among Saudi university students, while lack of time and other behavioral constraints have emerged as important barriers to regular activity (7–9). However, evidence examining whether differences in physical activity are independently associated with HRQoL in Saudi academic settings remains limited. Furthermore, much of the available university-based research focuses specifically on students, while HRQoL research involving university employees has often examined staff separately (12–14). Consequently, there is less evidence evaluating physical activity and HRQoL across students, faculty, and administrative staff within the same institutional setting, despite their differing academic and occupational demands. Examining these groups within the same institutional setting therefore provides an opportunity to assess physical activity and HRQoL across the broader university community while accounting for relevant sociodemographic and health-related differences in the multivariable analyses.

Therefore, this study aimed to examine physical activity, HRQoL, and perceived barriers to physical activity among students, faculty, and staff at Alfaisal University in Riyadh, Saudi Arabia. Specifically, it assessed physical activity patterns across the university community, examined the association between physical activity and HRQoL after accounting for relevant sociodemographic and health-related factors, and evaluated perceived barriers to physical activity. By examining these components within a single academic population, the study sought to provide evidence that may inform health promotion and well-being initiatives in university settings.

## 2 Materials and methods

### 2.1 Study design and setting

A cross-sectional study was conducted at Alfaisal University in Riyadh, Saudi Arabia, between October 2024 and April 2025, to examine physical activity, health-related quality of life (HRQoL), and perceived barriers to physical activity among members of the university community. Participant data were collected in January 2025. The study population comprised undergraduate and graduate students, faculty members, and administrative staff. During the 2023–2024 academic year, the university community included 5,669 undergraduate students, 682 graduate students, 371 faculty members, and 322 administrative staff.

### 2.2 Study population, sample size, and recruitment

A descriptive sample-size calculation for the university population of 7,044, assuming a 50% proportion, 95% confidence level, 5% absolute precision, and finite-population correction, yielded a recruitment benchmark of approximately 365 participants. This calculation was not designed to establish power for the multivariable association analyses or comparisons between university roles. Participants were recruited by convenience sampling, with efforts to include students, faculty, and administrative staff so that their characteristics could be described separately. The nominal 5% precision should therefore not be interpreted as an achieved margin of error for the university population, and comparisons between roles were considered exploratory.

Participants were recruited using convenience sampling through university email, internal communication platforms, and flyers distributed across the campus. The questionnaire was administered electronically through a quick response (QR) code. To minimize duplicate submissions, participants completed the questionnaire individually under the supervision of the principal investigator at the time of recruitment. Participation was anonymous, and no personal identifiers were collected. Because recruitment included open university-wide electronic communications and campus flyers, the total number of individuals who viewed or received the invitation could not be determined; therefore, a conventional response rate could not be calculated.

Individuals were eligible to participate if they were aged ≥18 years, were currently enrolled or employed at Alfaisal University, and provided informed consent. Individuals younger than 18 years, those not affiliated with the university, those unable or unwilling to provide consent, those with medical conditions preventing physical activity, and those with incomplete or inconsistent questionnaire responses were excluded. This criterion was intended to distinguish low physical activity from a medical inability to engage in physical activity. A pre-existing physical or mental health condition alone was not a reason for exclusion.

### 2.3 Data collection and study measures

Data were collected using a standardized self-administered questionnaire incorporating three validated instruments: the International Physical Activity Questionnaire–Short Form (IPAQ-SF) to assess physical activity, the EQ-5D-5L and EQ-VAS to assess HRQoL, and the Barriers to Being Active Quiz to assess perceived barriers to physical activity. Sociodemographic and health-related information was also collected for use in descriptive and multivariable analyses. The questionnaire was administered in English only, and no translation or linguistic adaptation was performed. English was used because it is the primary language of instruction at Alfaisal University, and students are required to demonstrate English proficiency for admission.

### 2.4 Physical activity assessment

Physical activity during the preceding 7 days was assessed using the International Physical Activity Questionnaire–Short Form (IPAQ-SF) (15). The instrument captures walking, moderate-intensity activity, vigorous-intensity activity, and sitting time. Physical activity was quantified using metabolic equivalent of task minutes per week (MET-min/week) and classified as low, moderate, or high according to standardized IPAQ scoring criteria (15). Walking, moderate-intensity activity, and vigorous-intensity activity were assigned values of 3.3, 4.0, and 8.0 METs, respectively, for calculation of MET-min/week.

Before scoring the IPAQ-SF, reported durations were converted to minutes, and activity lasting less than 10 min was not counted. Reported daily durations exceeding 180 min were truncated to 180 min separately for walking, moderate-intensity activity, and vigorous-intensity activity. The resulting values were used to calculate weekly activity and classify participants into low, moderate, and high physical activity categories. Sitting time was analyzed separately as a continuous variable in additional analyses examining its association with HRQoL outcomes.

### 2.5 Health-related quality of life assessment

HRQoL was assessed using the EQ-5D-5L and EQ-VAS (16). The EQ-5D-5L evaluates health across five dimensions: mobility, self-care, usual activities, pain/discomfort, and anxiety/depression, with each dimension rated across five levels of severity. The EQ-VAS records participants’ self-rated overall health on a scale from 0, representing the worst imaginable health, to 100, representing the best imaginable health.

HRQoL was analyzed using both the EQ-5D-5L index score and EQ-VAS score. The five individual EQ-5D-5L dimensions were additionally examined to evaluate associations with physical activity and other participant characteristics. Each participant’s responses across the five EQ-5D-5L dimensions were combined into a five-digit health state. Index scores were calculated by applying the Saudi Arabian EQ-5D-5L value set published by Al-Jedai et al. (17) to each health state. Because of sparse responses and model convergence issues within the self-care dimension, responses were dichotomized as no problems versus any problems for regression analysis.

### 2.6 Perceived barriers to physical activity

Perceived barriers were assessed using the 21-item Barriers to Being Active Quiz (18), which evaluates seven barrier domains: lack of time, social influence, lack of energy, lack of willpower, fear of injury, lack of skill, and lack of resources. Each domain comprises three items scored from 0 to 3, with a domain score of ≥5 indicating a significant perceived barrier. Internal consistency was assessed separately for each three-item barrier domain using Cronbach’s alpha. Barrier domains were analyzed as continuous scores and summarized using medians and interquartile ranges.

### 2.7 Statistical analysis

Descriptive statistics were used to summarize participant characteristics, physical activity, HRQoL, and perceived barriers. Physical activity was classified into low, moderate, and high categories according to IPAQ-SF scoring criteria. Ordinal logistic regression was used to examine factors associated with physical activity category. In this physical-activity model, age group, level of education, and monthly income were entered as ordered category scores, while university role was represented by separate categorical indicators. The odds ratios for the ordered predictors represent a one-category increase and assume a constant change in log odds across successive categories. This coding limited the number of coefficients estimated in the model. The proportional-odds assumption was assessed before fitting the ordinal logistic regression model. As the assumption was met, a standard proportional-odds model was used.

As a supplementary analysis, binary logistic regression examined factors associated with achieving ≥150 min of physical activity per week (≥150 min = 1; <150 min = 0), adjusting for age, gender, nationality, education level, university role, marital status, monthly income, and pre-existing physical and mental health conditions. Separate multiple linear regression models examined EQ-VAS and EQ-5D-5L index scores. Physical activity level, age group, education level, university role, and monthly income were entered as categorical indicator variables. Their reference categories were low physical activity, age 18–24 years, high school education, staff/administration, and no income, respectively. Gender, nationality, marital status, and pre-existing physical and mental health conditions were entered as binary variables; the marital-status comparison was married versus all other marital statuses. Unstandardized coefficients were estimated using SPSS Statistics version 30. Bias-corrected and accelerated (BCa) 95% confidence intervals were calculated separately in Python using 5,000 case-resampling bootstrap samples; the reported two-sided bootstrap *p*-values were obtained by inverting the BCa confidence procedure.

Associations between physical activity and the individual EQ-5D-5L dimensions were examined using ordinal logistic regression for mobility, usual activities, pain/discomfort, and anxiety/depression. Self-care was analyzed using binary logistic regression because of sparse data. Multivariable models examining the association between physical activity and HRQoL were adjusted for age, gender, nationality, education level, university role, marital status, monthly income, and pre-existing physical and mental health conditions.

As a sensitivity analysis addressing the bounded EQ-5D-5L index and its concentration at 1.0, we fitted a fractional logistic regression model with the same covariates and reference categories as the primary index model. All 353 index observations were retained. HC3 robust standard errors were used, and adjusted average differences in index score between physical activity categories were estimated from model predictions.

Barrier scores were summarized using medians and interquartile ranges. Separate multivariable linear regression models examined the association between physical activity level and each barrier score. Physical activity level was entered as an ordinal score (low, moderate, high), and the models were adjusted for age, gender, and university role. Fear of injury and lack of skill scores were log-transformed because of non-normal residual distributions, and bootstrap confidence intervals were used for these outcomes. Model diagnostics included assessment of multicollinearity using variance inflation factors, examination of normality and linearity of residuals for linear regression models, and assessment of the proportional odds assumption for ordinal models. Analyses were performed using IBM SPSS Statistics version 30, except for the BCa bootstrap inference for the primary EQ-VAS and EQ-5D-5L models and the fractional logistic sensitivity analysis, which were performed in Python.

### 2.8 Ethical considerations

The study protocol was approved by the Institutional Review Board of Alfaisal University (IRB approval No. IRB20346). Participation was voluntary, and electronic informed consent was obtained before participants accessed the questionnaire. Participants were informed of the study purpose, confidentiality procedures, voluntary nature of participation, and their right to withdraw without consequence. Responses were collected anonymously, and no personally identifiable information was recorded.

## 3 Results

### 3.1 Participant characteristics

Of the 365 responses received, 12 were excluded based on the study’s eligibility or data-quality criteria, leaving 353 participants in the final analysis, comprising students, faculty, and staff/administrative employees. No responses were excluded because of a medical condition preventing physical activity. Of the 353 participants included, there were 288 students (81.6%), 40 faculty members (11.3%), and 25 staff/administrative employees (7.1%). Most were aged 18–24 years (242, 68.6%), and 202 (57.2%) were female. Pre-existing physical and mental health conditions were reported by 54 (15.3%) and 30 (8.5%) participants, respectively. Full participant characteristics by university role are presented in Supplementary Table S1. Physical activity characteristics by university role are presented in Supplementary Table S2.

### 3.2 Physical activity patterns

Moderate physical activity was the most frequently reported IPAQ-SF category (43.6%), followed by high (28.6%) and low (27.8%) physical activity. Among the participants, the median was 1,618.5 MET-min/week (IQR 2,870.75; range 0–15,750). Forty-seven participants (13.4%) had a total of 0 MET-min/week. Physical activity patterns varied across participant characteristics and university roles. Sitting time was available for 217 participants. The mean was 423.2 min/day (SD 237.3), and the median was 360 min/day (IQR 255); values ranged from 0 to 1,200 min/day. One participant (0.5%) reported 0 min/day.

The proportional-odds assumption was met; therefore, no threshold-specific coefficients were estimated. In the adjusted ordinal logistic regression model, female participants had higher odds of being in a lower physical activity category compared with male participants (aOR = 1.897, 95% CI: 1.267–2.840, *p* = 0.002). Saudi participants had lower odds of being in a lower physical activity category than non-Saudi participants (adjusted OR = 0.589, 95% CI: 0.396–0.875, *p* = 0.009). Age, education level, university role, marital status, monthly income, and pre-existing physical and mental health conditions were not significantly associated with physical activity category after adjustment. The complete adjusted model is presented in Table 1.

**Table 1:** Adjusted sociodemographic and health-related predictors of physical activity level (*N* = 353).

Predictor	Adjusted OR	95% CI	*p*-value
Age	0.700	0.456–1.540	0.088
Gender (female vs. male)	**1.897**	**1.267–2.840**	**0.002**
Nationality (Saudi vs. non-Saudi)	**0.589**	**0.396–0.875**	**0.009**
Level of education	1.420	0.987–2.050	0.059
Role: student vs. staff/administration	0.610	0.257–1.449	0.263
Role: faculty vs. staff/administration	0.934	0.349–2.504	0.893
Marital status (married vs. other marital statuses)	1.041	0.439–2.467	0.928
Monthly income (SAR)	0.898	0.760–1.063	0.211
Pre-existing physical condition (yes vs. no)	1.413	0.822–2.430	0.211
Pre-existing mental health condition (yes vs. no)	1.974	0.977–3.987	0.058

OR, odds ratio; CI, confidence interval; SAR, Saudi Arabian riyal. Physical activity was modeled as an ordinal outcome (low, moderate, high). An adjusted OR >1 indicates higher odds of being in a lower physical activity category, whereas an adjusted OR <1 indicates lower odds of being in a lower physical activity category. Reference categories were male gender, non-Saudi nationality, staff/administration role, all other marital statuses, and absence of pre-existing physical and mental health conditions. For age group, level of education, and monthly income, adjusted odds ratios are reported per one-category increase. Bold values indicate statistical significance (*p* < 0.05).

In the supplementary binary logistic regression analysis, the overall model for achieving ≥150 min of physical activity per week was statistically significant (χ^2^(19) = 50.991, *p* < 0.001; Nagelkerke R^2^ = 0.186). Female participants had lower odds of achieving this threshold than male participants (adjusted OR = 0.452, 95% CI: 0.271–0.755, *p* = 0.002). Participants aged 35–44 years also had lower odds than those aged 18–24 years (adjusted OR = 0.050, 95% CI: 0.007–0.369, *p* = 0.003). Full results are presented in Supplementary Table S3.

### 3.3 Physical activity and health-related quality of life

EQ-VAS scores were available for 352 participants. The mean was 78.9 (SD 19.9), and the median was 85 (IQR 21); scores ranged from 0 to 100. Forty participants (11.4%) scored 100, and two (0.6%) scored 0. The mean EQ-5D-5L index score was 0.928 (SD 0.107), and the median was 0.961 (IQR 0.111); observed scores ranged from 0.343 to 1.000. A total of 138 of 353 participants (39.1%) reported the full-health state (11111) and had the maximum index score of 1.000.

The EQ-VAS model included 352 participants, and the EQ-5D-5L index model included 353. After adjustment for sociodemographic and health-related factors, physical activity level was not significantly associated with EQ-VAS. Compared with low physical activity, the coefficient was 1.761 for moderate physical activity (BCa 95% CI: −3.620 to 7.500, *p* = 0.572) and 4.055 for high physical activity (BCa 95% CI: −2.082 to 10.426, *p* = 0.197).

Physical activity level was also not significantly associated with EQ-5D-5L index scores. Compared with low physical activity, the coefficient was −0.016 for moderate physical activity (BCa 95% CI: −0.039 to 0.011, *p* = 0.231) and −0.005 for high physical activity (BCa 95% CI: −0.035 to 0.028, *p* = 0.824).

The EQ-5D-5L index model residuals departed from normality (Shapiro–Wilk W = 0.797, *p* < 0.001), and six fitted values from the linear model exceeded the maximum possible index score of 1.0. In the fractional logistic sensitivity analysis, moderate physical activity was associated with an adjusted average index difference of −0.017 compared with low physical activity (robust 95% CI: −0.042 to 0.007, *p* = 0.159); the corresponding difference for high physical activity was −0.007 (robust 95% CI: −0.037 to 0.023, *p* = 0.657). The physical activity terms were not jointly significant (*p* = 0.377).

Consistent with these findings, adjusted analyses of the individual EQ-5D-5L dimensions showed no statistically significant association between physical activity level and mobility, self-care, usual activities, pain/discomfort, or anxiety/depression (Supplementary Table S4).

Sitting time was also not significantly associated with HRQoL after adjustment. Each additional minute of daily sitting time was associated with an EQ-VAS coefficient of −0.009 (BCa 95% CI: −0.024 to 0.008, *p* = 0.240), while the association with the EQ-5D-5L index was approximately zero (*p* = 0.864). The full adjusted sitting-time results are presented in Supplementary Table S5.

### 3.4 Factors associated with health-related quality of life

In the EQ-VAS model, a pre-existing mental health condition was associated with a lower score (B = −11.891, BCa 95% CI: −22.157 to −2.653, *p* = 0.010). Female participants had a higher adjusted score than male participants (B = 4.333, BCa 95% CI: 0.162 to 8.501, *p* = 0.041). Compared with participants aged 18–24 years, those aged 25–34 years had a higher adjusted score (B = 9.090, BCa 95% CI: 1.943 to 16.863, *p* = 0.011). Participants with a bachelor’s degree had a lower adjusted score than those with a high school education (B = −7.887, BCa 95% CI: −14.900 to −1.684, *p* = 0.010).

In the EQ-5D-5L index model, pre-existing mental and physical health conditions were associated with lower scores (mental condition: B = −0.088, BCa 95% CI: −0.155 to −0.034, *p* = 0.001; physical condition: B = −0.042, BCa 95% CI: −0.083 to −0.009, *p* = 0.013). Married participants had a higher adjusted index score than other participants (B = 0.044, BCa 95% CI: 0.003 to 0.083, *p* = 0.040), as did participants aged 25–34 years compared with those aged 18–24 years (B = 0.033, BCa 95% CI: 0.001 to 0.073, *p* = 0.043). Full results for both models are presented in Table 2.

**Table 2:** Adjusted associations of physical activity and participant characteristics with HRQoL.

Predictor	EQ-VAS B (BCa 95% CI)	*p*-value	EQ-5D-5L index B (BCa 95% CI)	*p*-value
Moderate vs. low PA	1.761 (−3.620 to 7.500)	0.572	−0.016 (−0.039 to 0.011)	0.231
High vs. low PA	4.055 (−2.082 to 10.426)	0.197	−0.005 (−0.035 to 0.028)	0.824
Female vs. male	4.333 (0.162 to 8.501)	**0.041**	−0.016 (−0.038 to 0.005)	0.145
Non-Saudi vs. Saudi	1.381 (−3.178 to 6.116)	0.556	0.012 (−0.011 to 0.038)	0.336
Married vs. other marital status	6.198 (−1.029 to 14.268)	0.087	0.044 (0.003 to 0.083)	**0.040**
Pre-existing physical condition	−4.496 (−10.802 to 1.193)	0.123	−0.042 (−0.083 to −0.009)	**0.013**
Pre-existing mental condition	−11.891 (−22.157 to −2.653)	**0.010**	−0.088 (−0.155 to −0.034)	**0.001**
Age: 25–34	9.090 (1.943 to 16.863)	**0.011**	0.033 (0.001 to 0.073)	**0.043**
Age: 35–44	−3.658 (−16.504 to 8.243)	0.529	−0.018 (−0.098 to 0.042)	0.559
Age: 45–54	0.580 (−12.463 to 13.566)	0.958	−0.074 (−0.202 to 0.028)	0.186
Age: 55+	−0.090 (−13.939 to 14.926)	0.983	−0.058 (−0.174 to 0.034)	0.226
Education: Bachelor	−7.887 (−14.900 to −1.684)	**0.010**	−0.008 (−0.045 to 0.024)	0.631
Education: Master	−7.640 (−18.133 to 1.414)	0.105	−0.003 (−0.049 to 0.041)	0.878
Education: PhD	−6.451 (−18.695 to 3.756)	0.192	0.019 (−0.054 to 0.093)	0.605
Role: Student	−0.114 (−9.027 to 9.235)	0.937	−0.031 (−0.067 to 0.006)	0.108
Role: Faculty	−0.477 (−9.711 to 8.924)	0.879	−0.016 (−0.053 to 0.024)	0.436
Income: <=4,999	0.506 (−6.625 to 7.242)	0.858	−0.012 (−0.063 to 0.023)	0.528
Income: 5000–9,999	4.984 (−6.156 to 12.645)	0.333	−0.014 (−0.057 to 0.020)	0.398
Income: 10000–14,999	3.599 (−6.298 to 12.853)	0.447	−0.014 (−0.067 to 0.029)	0.527
Income: 15000–19,999	2.379 (−14.219 to 13.815)	0.705	0.000 (−0.046 to 0.046)	0.994
Income: 20000+	4.570 (−2.357 to 12.190)	0.183	0.004 (−0.031 to 0.040)	0.846

B, unstandardized regression coefficient; BCa CI, bias-corrected and accelerated bootstrap 95% confidence interval based on 5,000 case-resampling samples; EQ-VAS, EuroQol Visual Analog Scale; SAR, Saudi Arabian riyal. Two-sided p-values were obtained by inverting the BCa confidence procedure. The EQ-VAS model included 352 participants (R^2^ = 0.105; adjusted R^2^ = 0.048), and the EQ-5D-5L index model included 353 (R^2^ = 0.148; adjusted R^2^ = 0.094). Reference categories were low physical activity, male, Saudi, all marital statuses other than married, no pre-existing condition, age 18–24 years, high school education, staff/administration, and no income. Bold values indicate *p* < 0.05. Bold values indicate statistical significance (*p* < 0.05).

### 3.5 Perceived barriers to physical activity

Lack of willpower (67%), lack of energy (66%), and lack of time (65%) were the most frequently reported barriers to physical activity. Lack of resources (43%) and social influence (42%) were also commonly reported, whereas lack of skill (24%) and fear of injury (16%) were less frequently reported (Figure 1).

**Figure 1:**
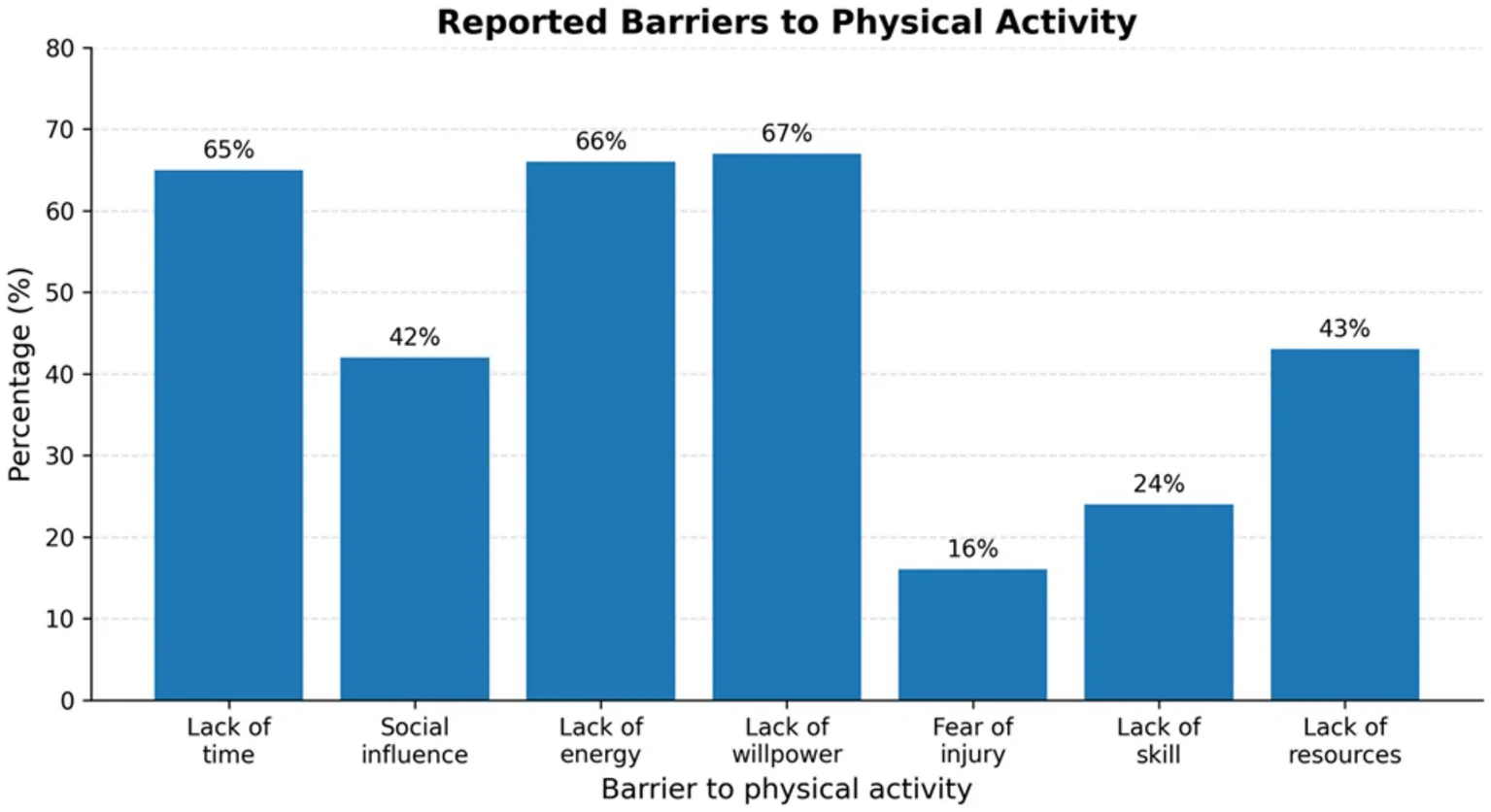
Reported barriers to physical activity among study participants. Percentage of participants reporting each perceived barrier to physical activity. Lack of willpower (67%), lack of energy (66%), and lack of time (65%) were the most frequently reported barriers.

Cronbach’s alpha was 0.819 for lack of time, 0.633 for social influence, 0.782 for lack of energy, 0.818 for lack of willpower, 0.864 for fear of injury, 0.845 for lack of skill, and 0.607 for lack of resources (*n* = 353 for each domain).

After adjustment for age, gender, and university role, each one-category increase in physical activity level was associated with lower lack-of-willpower scores (B = −0.711, 95% CI: −1.088 to −0.333, *p* < 0.001) and lack-of-time scores (B = −0.608, 95% CI: −0.962 to −0.255, *p* < 0.001). Associations with social influence, lack of energy, and lack of skill were also negative, whereas the confidence intervals for fear of injury and lack of resources included zero. Adjusted coefficients and confidence intervals for all seven barrier domains are presented in Table 3. The lack-of-skill coefficient is on the log-transformed score scale.

**Table 3:** Adjusted associations between physical activity level and perceived barrier scores.

Barrier outcome	B	95% CI	*p*-value
Lack of time	**−0.608**	**−0.962 to −0.255**	**<0.001**
Social influence	**−0.412**	**−0.740 to −0.085**	**0.014**
Lack of energy	**−0.464**	**−0.824 to −0.104**	**0.012**
Lack of willpower	**−0.711**	**−1.088 to −0.333**	**<0.001**
Fear of injury*	0.049	−0.060 to 0.159	0.375
Lack of skill*	**−0.119**	**−0.231 to −0.006**	**0.039**
Lack of resources	−0.041	−0.394 to 0.312	0.820

B, unstandardized regression coefficient; CI, confidence interval. Physical activity level was entered as an ordinal variable (low, moderate, high), with higher values representing greater physical activity. Models were adjusted for age, gender, and university role. Negative coefficients indicate that higher physical activity level was associated with lower perceived barrier scores. *Fear of injury and lack of skill were log-transformed because of non-normal residual distributions; bootstrapped coefficients and confidence intervals are reported. Bold values indicate *p* < 0.05. Bold values indicate statistical significance (*p* < 0.05).

## 4 Discussion

### 4.1 Principal findings

This study examined physical activity, health-related quality of life (HRQoL), and perceived barriers to physical activity among students, faculty, and staff at Alfaisal University. The principal finding was that no statistically significant independent association between physical activity level and HRQoL was detected after adjustment for sociodemographic and health-related factors. No statistically significant associations were detected with either EQ-VAS or EQ-5D-5L index scores, and no significant associations were observed between physical activity and the individual EQ-5D-5L dimensions. Sitting time was also not independently associated with HRQoL. In contrast, pre-existing health conditions, particularly mental health conditions, were associated with poorer HRQoL. Female participants had higher odds of being in a lower physical activity category than male participants, whereas Saudi participants had lower odds of being in a lower physical activity category than non-Saudi participants. Lack of time, energy, and willpower emerged as the most prominent perceived barriers to physical activity. Together, these findings suggest that HRQoL within this university community reflects multiple physical, psychological, and behavioral influences rather than physical activity level alone.

### 4.2 Physical activity and health-related quality of life

The absence of an independent association between physical activity and HRQoL differs from much of the existing literature reviewed in this study. Previous international evidence has generally demonstrated positive associations between physical activity and HRQoL across different populations. Nguyen et al. (10) reported improvements in general and mental HRQoL associated with physical exercise among office workers, with particularly notable benefits among individuals with pre-existing health conditions. Positive associations have also been reported among university students, including evidence that more physically active students report better quality-of-life outcomes (11). Similar findings have been reported in Saudi Arabia, where Abdulrashid et al. (19) found that higher physical activity was associated with better physical and mental HRQoL among adults in Jeddah. However, that study assessed physical activity using the General Practice Physical Activity Questionnaire (GPPAQ) and HRQoL using the SF-36, whereas the present study used the IPAQ-SF and EQ-5D-5L. Differences in study populations, measurement instruments, covariate adjustment, and analytical approaches may therefore contribute to differences in findings across studies.

Several characteristics of the present study may help explain why an independent association was not detected. Participants were largely young and reported generally favorable HRQoL, while most were classified as having moderate or high physical activity. In addition, 39.1% reported the full-health state (11111) and had the maximum EQ-5D-5L index score, indicating a ceiling effect. This concentration at the top of the index may have limited its ability to distinguish differences in HRQoL among healthier participants and may have reduced sensitivity to an association with physical activity.

The absence of a statistically significant association should not be interpreted as evidence that physical activity is unrelated to HRQoL. Rather, the present study did not detect a statistically significant independent association between physical activity level and HRQoL within this academic population after accounting for measured sociodemographic and health-related characteristics. Given the cross-sectional design, self-reported measures, and potential for residual confounding, the findings should be interpreted within the context of the present sample and study design. This cautious interpretation is consistent with the broader literature demonstrating the established physical and psychological health benefits of regular physical activity (3).

The secondary analysis of sitting time produced a similar pattern, with no significant independent association with either EQ-VAS or EQ-5D-5L index scores. This contrasts with previous university-based evidence reporting associations between prolonged sitting and poorer aspects of HRQoL. For example, García-Pérez-de-Sevilla et al. (14) reported an inverse association between sitting time and physical functioning among healthy university students despite relatively high adherence to physical activity recommendations. The present findings therefore reinforce the possibility that HRQoL within a relatively healthy academic population is influenced by a combination of behavioral and health-related factors rather than physical activity or sedentary behavior in isolation.

### 4.3 Health status and health-related quality of life

In contrast to physical activity, pre-existing health conditions showed clearer associations with HRQoL. Pre-existing mental health conditions were associated with lower EQ-VAS and EQ-5D-5L index scores, while pre-existing physical health conditions were also associated with poorer EQ-5D-5L index scores. These findings are consistent with previous evidence demonstrating the substantial contribution of psychological and physical health status to perceived quality of life in academic and general populations (12, 13).

The individual EQ-5D-5L dimensions further illustrate the multidimensional nature of these relationships. These findings are consistent with conceptual models of HRQoL in which physical functioning, psychological health, symptoms, and everyday functioning interact to shape an individual’s overall perception of health.

The association between mental health conditions and multiple HRQoL outcomes is particularly relevant in an academic environment. Previous research has reported associations between depression, anxiety, psychological strain, chronic health problems, and poorer HRQoL among students and university employees (12, 13). The present findings extend this perspective to a university community incorporating students, faculty, and staff. However, given the cross-sectional design, the direction and causality of these associations cannot be established, and residual or unmeasured confounding may have contributed to the observed relationships. Nevertheless, the findings highlight the importance of considering psychological health alongside lifestyle behaviors such as physical activity when examining well-being within academic institutions.

### 4.4 Physical activity patterns and perceived barriers

Female participants had higher odds of being in a lower physical activity category than male participants after adjustment. Gender differences in physical activity have also been reported in university populations internationally and within Saudi Arabia (8, 9), although the direction and magnitude of these differences have varied across studies. These differences may reflect a combination of behavioral, environmental, social, and contextual influences on opportunities for physical activity. Given the cross-sectional nature of the present study, however, the mechanisms underlying the observed gender difference cannot be determined.

Lack of time, lack of energy, and lack of willpower were the most prominent barriers across university roles. This pattern is consistent with previous research in university populations identifying academic demands, competing priorities, and motivational factors as important obstacles to physical activity. Similar barriers have been reported in Saudi populations, including lack of time, low motivation, academic demands, limited access to facilities, environmental constraints, and cultural or social factors (20, 21).

Higher physical activity levels were also associated in the present analysis with lower perceived barriers, particularly those related to time and willpower. Because these data are cross-sectional, the direction of these relationships cannot be established: fewer perceived barriers may facilitate greater physical activity, while individuals who are regularly active may also perceive or manage such barriers differently. The findings nevertheless indicate potentially modifiable factors that may be relevant when designing university-based physical activity initiatives.

### 4.5 Public health implications

The absence of an independent association between physical activity and HRQoL in this study does not diminish the established importance of physical activity as a public health priority (3). Instead, the findings suggest that university health promotion may benefit from a broader approach to well-being. Interventions focused exclusively on increasing physical activity may overlook psychological, behavioral, and environmental factors that also contribute to perceived health and participation in active lifestyles.

University-based initiatives could therefore combine accessible and flexible opportunities for physical activity with strategies addressing commonly reported barriers, particularly lack of time, energy, and motivation. Integration with mental health and wellness services may be particularly relevant given the consistent association between pre-existing mental health conditions and poorer HRQoL observed in this study. Such a multidimensional approach is also consistent with broader health-promotion objectives in Saudi Arabia that emphasize active lifestyles, prevention of noncommunicable diseases, and population well-being under Saudi Vision 2030 (5).

### 4.6 Strengths and limitations

This study has several strengths. It examined physical activity and HRQoL within a single analytical framework among students, faculty, and staff, providing a broader perspective on the university community than studies restricted to students alone. Validated instruments were used to assess physical activity, HRQoL, and perceived barriers, including the IPAQ-SF, EQ-5D-5L, EQ-VAS, and Barriers to Being Active Quiz. The analyses also considered multiple sociodemographic and health-related factors, and bias-corrected and accelerated bootstrapping was applied where appropriate to improve the robustness of regression estimates. In addition to physical activity level, the study considered sitting time and perceived barriers, providing a more comprehensive assessment of activity-related behaviors.

Several limitations should be considered when interpreting the findings. First, the cross-sectional design prevents determination of temporal or causal relationships between physical activity and HRQoL. Second, convenience sampling may have introduced selection bias and limits generalizability beyond the study population and similar university settings. Because the questionnaire was available only in English and individual language proficiency was not assessed, language-related differences in participation or comprehension remain possible. Moreover, because a conventional response rate could not be calculated, non-response bias cannot be excluded; additionally, supervised questionnaire completion may have influenced participants’ responses despite anonymous data collection. Although no responses were excluded under the criterion concerning medical conditions preventing physical activity, the findings may not apply to people who are medically unable to be active. Third, physical activity was self-reported and may therefore be affected by recall and social desirability bias; objective measures such as accelerometers were not available. Sitting time could be quantified for only 217 of 353 participants (61.5%), which limits interpretation of the sitting-time analyses. Fourth, the relatively young population, generally favorable HRQoL, and high prevalence of moderate-to-high physical activity may have resulted in restricted variability and reduced the ability to identify associations. The substantial ceiling effect and non-normal residuals also limit the fit of the linear model for the EQ-5D-5L index. A fractional logistic sensitivity analysis produced the same qualitative conclusion regarding physical activity. Fifth, internal consistency was lower for the social influence and lack of resources barrier domains, so findings for these two domains should be interpreted cautiously. Sixth, the sample-size calculation was intended for descriptive estimation rather than to establish power for the adjusted analyses. The confidence intervals do not rule out potentially relevant associations between physical activity and HRQoL, and the small faculty and staff groups limit conclusions from comparisons by university role. Treating age group, education level, and income as ordered scores in the physical-activity model assumes a constant trend across successive categories; this may not fully capture differences between individual categories. Finally, although the multivariable analyses adjusted for several relevant sociodemographic and health-related factors, residual confounding cannot be excluded.

### 4.7 Future research

Future studies should use longitudinal designs to clarify the temporal relationship between physical activity and HRQoL in university populations. Objective assessment of physical activity and sedentary behavior using wearable devices or accelerometers could complement self-reported measures and reduce measurement bias. Studies involving larger and more diverse university populations across multiple institutions would also improve generalizability and permit more detailed comparisons among students, faculty, and staff. Further research should examine how mental health, physical health, perceived barriers, and physical activity interact over time and whether integrated university-based interventions can improve both activity participation and broader measures of well-being.

## 5 Conclusion

Physical activity level was not independently associated with HRQoL among students, faculty, and staff at Alfaisal University after adjustment for sociodemographic and health-related factors. In contrast, pre-existing health conditions, particularly mental health conditions, were consistently associated with poorer HRQoL. Lack of time, energy, and willpower were the most prominent perceived barriers to physical activity, highlighting potentially modifiable challenges within the university environment. These findings suggest that university health promotion should extend beyond promoting physical activity alone and adopt a more integrated approach that addresses mental well-being and barriers to active lifestyles. Longitudinal and multicenter studies incorporating objective measures of physical activity are needed to further clarify the relationship between physical activity and HRQoL in university populations.

## Data Availability

The raw data supporting the conclusions of this article will be made available by the authors, without undue reservation.
