# Supplementary material for: Physical activity, health-related quality of life, and perceived barriers among the Alfaisal University Community in Riyadh, Saudi Arabia: a cross-sectional study
Source: Front Public Health. 2026 Oct 23;14:1999364. doi: 10.3389/fpubh.2026.1999364 (PMC13761317; doi:10.3389/fpubh.2026.1999364)
Supplement: Supplementary file 1 [file Table_1.docx]

Supplementary Material

# 1 Supplementary Tables

**Supplementary Table S1. Participant characteristics by university role (N = 353).**

| Characteristic | Students (n = 288) | Faculty (n = 40) | Staff/Administration (n = 25) |
| --- | --- | --- | --- |
| Age, years |  |  |  |
| 18–24 | 236 (81.9%) | 3 (7.5%) | 3 (12.0%) |
| 25–34 | 44 (15.3%) | 20 (50.0%) | 14 (56.0%) |
| 35–44 | 7 (2.4%) | 7 (17.5%) | 1 (4.0%) |
| 45–54 | 0 (0.0%) | 5 (12.5%) | 6 (24.0%) |
| ≥55 | 1 (0.3%) | 5 (12.5%) | 1 (4.0%) |
| Gender |  |  |  |
| Male | 125 (43.4%) | 18 (45.0%) | 8 (32.0%) |
| Female | 163 (56.6%) | 22 (55.0%) | 17 (68.0%) |
| Marital status |  |  |  |
| Single | 276 (95.8%) | 20 (50.0%) | 20 (80.0%) |
| Married | 12 (4.2%) | 20 (50.0%) | 5 (20.0%) |
| Nationality |  |  |  |
| Saudi | 136 (47.2%) | 5 (12.5%) | 10 (40.0%) |
| Non-Saudi | 152 (52.8%) | 35 (87.5%) | 15 (60.0%) |
| Education level |  |  |  |
| High school diploma or equivalent | 175 (60.8%) | 0 (0.0%) | 1 (4.0%) |
| Bachelor’s degree | 90 (31.3%) | 16 (40.0%) | 19 (76.0%) |
| Master’s degree | 20 (6.9%) | 11 (27.5%) | 4 (16.0%) |
| Doctorate (PhD) | 3 (1.0%) | 13 (32.5%) | 1 (4.0%) |
| Monthly income (SAR) |  |  |  |
| No income | 185 (64.2%) | 0 (0.0%) | 2 (8.0%) |
| ≤4,999 | 38 (13.2%) | 3 (7.5%) | 1 (4.0%) |
| 5,000–9,999 | 19 (6.6%) | 15 (37.5%) | 11 (44.0%) |
| 10,000–14,999 | 18 (6.3%) | 3 (7.5%) | 5 (20.0%) |
| 15,000–19,999 | 7 (2.4%) | 8 (20.0%) | 4 (16.0%) |
| ≥20,000 | 21 (7.3%) | 11 (27.5%) | 2 (8.0%) |
| Pre-existing physical condition |  |  |  |
| No | 245 (85.1%) | 34 (85.0%) | 20 (80.0%) |
| Yes | 43 (14.9%) | 6 (15.0%) | 5 (20.0%) |
| Pre-existing mental health condition |  |  |  |
| No | 260 (90.3%) | 39 (97.5%) | 24 (96.0%) |
| Yes | 28 (9.7%) | 1 (2.5%) | 1 (4.0%) |

Data are presented as n (%). Percentages are calculated within university-role groups. SAR, Saudi Arabian riyal.

## Supplementary Table S2. Physical activity characteristics by university role

| Physical activity characteristic | Students (n=288) | Faculty (n=40) | Staff/Administration (n=25) |
| --- | --- | --- | --- |
| IPAQ-SF physical activity level, n (%) |  |  |  |
| Low | 86 (29.9%) | 6 (15.0%) | 6 (24.0%) |
| Moderate | 116 (40.2%) | 25 (62.5%) | 13 (52.0%) |
| High | 86 (29.9%) | 9 (22.5%) | 6 (24.0%) |
| Physical activity duration, median (IQR), min/week |  |  |  |
| Vigorous activity | 180 (260) | 232 (187) | 300 (780) |
| Moderate activity | 127.5 (300) | 137 (180) | 130 (452) |
| Walking | 240 (480) | 360 (630) | 270 (647) |
| Total activity | 450 (668.75) | 450 (645) | 390 (810) |
| Sitting time, median (IQR), min/day |  |  |  |
| Sitting^a^ | 360 (279.75) | 360 (291.5) | 480 (330) |
| Physical activity, median (IQR), MET-min/week |  |  |  |
| Vigorous activity | 1,440 (2,080) | 1,860 (1,500) | 2,400 (6,240) |
| Moderate activity | 510 (1,200) | 550 (720) | 520 (1,810) |
| Walking | 792 (1,584) | 1,188 (2,079) | 891 (2,136) |

**Note.** Values are presented as n (%) within university role or median (IQR), as appropriate. IPAQ-SF, International Physical Activity Questionnaire–Short Form; IQR, interquartile range; MET-min/week, metabolic equivalent task minutes per week. Physical activity duration is reported in minutes per week and sitting time in minutes per day. ᵃ Sitting-time summaries include participants with available sitting-time data (n = 217 overall). Sitting time was not included in the MET-min/week total or physical activity classification.

**Supplementary Table S3. Multivariable binary logistic regression analysis of factors associated with sufficient physical activity (≥150 min/week)**

| Predictor | Category | Adjusted OR | 95% CI | p-value |
| --- | --- | --- | --- | --- |
| Age | 18–24 (reference) | — | — | — |
|  | 25–34 | 0.967 | 0.396–2.363 | 0.941 |
|  | 35–44 | **0.050** | **0.007–0.369** | **0.003** |
|  | 45–54 | 0.222 | 0.027–1.850 | 0.164 |
|  | ≥55 | 0.831 | 0.062–11.091 | 0.889 |
| Gender | Male (reference) | — | — | — |
|  | Female | **0.452** | **0.271–0.755** | **0.002** |
| Nationality | Saudi (reference) | — | — | — |
|  | Non-Saudi | 1.649 | 0.983–2.766 | 0.058 |
| Education | High school or equivalent (reference) | — | — | — |
|  | Bachelor’s degree | 0.963 | 0.515–1.800 | 0.905 |
|  | Master’s degree | 1.493 | 0.431–5.168 | 0.527 |
|  | Doctorate (PhD) | 0.657 | 0.111–3.901 | 0.644 |
| University role | Student (reference) | — | — | — |
|  | Faculty | 2.745 | 0.808–9.326 | 0.106 |
|  | Staff/Administration | 2.226 | 0.649–7.640 | 0.203 |
| Marital status | Single (reference) | — | — | — |
|  | Married | 2.269 | 0.580–8.876 | 0.239 |
| Monthly income (SAR) | No income (reference) | — | — | — |
|  | ≤4,999 | 2.361 | 0.953–5.847 | 0.063 |
|  | 5,000–9,999 | 0.602 | 0.243–1.491 | 0.273 |
|  | 10,000–14,999 | 0.748 | 0.246–2.279 | 0.610 |
|  | 15,000–19,999 | 0.331 | 0.074–1.481 | 0.148 |
|  | ≥20,000 | 0.651 | 0.219–1.936 | 0.440 |
| Pre-existing physical condition | No (reference) | — | — | — |
|  | Yes | 0.752 | 0.376–1.504 | 0.420 |
| Pre-existing mental health condition | No (reference) | — | — | — |
|  | Yes | 0.791 | 0.328–1.908 | 0.602 |

**Note.** The dependent variable was physical activity status, coded as insufficiently active (<150 min/week) = 0 and sufficiently active (≥150 min/week) = 1. OR, odds ratio; CI, confidence interval; SAR, Saudi Arabian riyal. Odds ratios were adjusted for all variables included in the model. The overall model was statistically significant, χ²(19) = 50.991, *p* < 0.001, with Nagelkerke R² = 0.186. Bold values indicate *p* < 0.05.

**Supplementary Table S4. Adjusted associations between physical activity level and individual EQ-5D-5L dimensions**

| EQ-5D-5L dimension | Physical activity level | Adjusted OR | 95% CI | p-value |
| --- | --- | --- | --- | --- |
| Mobility | Low | 1.117 | 0.327–3.812 | 0.860 |
|  | Moderate | 1.828 | 0.191–1.982 | 0.416 |
|  | High (reference) | — | — | — |
| Self-care | Low | 0.394 | 0.095–1.634 | 0.199 |
|  | Moderate | 0.616 | 0.218–2.261 | 0.553 |
|  | High (reference) | — | — | — |
| Usual activities | Low | 0.994 | 0.428–2.307 | 0.989 |
|  | Moderate | 0.985 | 0.450–2.158 | 0.970 |
|  | High (reference) | — | — | — |
| Pain/discomfort | Low | 0.664 | 0.356–1.241 | 0.199 |
|  | Moderate | 1.312 | 0.770–2.239 | 0.317 |
|  | High (reference) | — | — | — |
| Anxiety/depression | Low | 1.253 | 0.712–2.210 | 0.433 |
|  | Moderate | 1.467 | 0.886–2.428 | 0.136 |
|  | High (reference) | — | — | — |

**Note.** OR, odds ratio; CI, confidence interval; EQ-5D-5L, EuroQol 5-Dimension 5-Level instrument. High physical activity was the reference category. Models were adjusted for age, gender, nationality, education level, university role, marital status, and monthly income. No statistically significant associations were observed between physical activity level and any individual EQ-5D-5L dimension.

**Supplementary Table S5. Multiple linear regression with BCa bootstrapped confidence intervals for the association between sitting time and HRQoL outcomes**

| Predictor | EQ-VAS B | BCa 95% CI | p-value | EQ-5D-5L index B | BCa 95% CI | p-value |
| --- | --- | --- | --- | --- | --- | --- |
| Sitting time (min/day) | −0.009 | −0.024 to 0.008 | 0.240 | 0.000 | −0.000 to 0.000 | 0.864 |
| Sociodemographic factors |  |  |  |  |  |  |
| Age | 1.862 | −1.461 to 6.732 | 0.301 | −0.010 | −0.045 to 0.019 | 0.474 |
| Gender (ref: male) | 4.167 | −1.343 to 9.616 | 0.134 | −0.019 | −0.044 to 0.004 | 0.169 |
| Nationality | 2.607 | −3.727 to 9.375 | 0.414 | 0.016 | −0.011 to 0.045 | 0.296 |
| Level of education | −3.500 | −9.058 to 0.782 | 0.134 | 0.011 | −0.016 to 0.033 | 0.444 |
| University role | 1.031 | −3.556 to 5.236 | 0.641 | 0.010 | −0.016 to 0.032 | 0.396 |
| Marital status | 1.417 | −7.650 to 9.864 | 0.706 | 0.028 | −0.018 to 0.070 | 0.201 |
| Monthly income (SAR) | 1.636 | −0.414 to 3.429 | 0.150 | 0.001 | −0.009 to 0.012 | 0.935 |
| Health factors |  |  |  |  |  |  |
| Pre-existing physical condition | **−9.153** | **−17.048 to −1.405** | **0.015** | −0.032 | −0.076 to 0.019 | 0.164 |
| Pre-existing mental health condition | −5.312 | −16.743 to 4.582 | 0.373 | −0.054 | −0.122 to 0.022 | 0.173 |

**Note.** B, unstandardized regression coefficient; BCa CI, bias-corrected and accelerated bootstrap confidence interval based on 1,000 bootstrap samples; EQ-VAS, EuroQol Visual Analogue Scale; SAR, Saudi Arabian riyal. EQ-VAS scores range from 0–100 and EQ-5D-5L index scores ranged from 0.343 to 1.000. Sitting time was analyzed as a continuous predictor in minutes/day. Bold indicates *p* < 0.05.
